# Clinical Utility of Amyloid Imaging in a Complex Case of Corticobasal Syndrome Presenting with Psychiatric Symptoms

**DOI:** 10.4172/2329-6895.1000194

**Published:** 2014-11-26

**Authors:** MR Bensaïdane, Fortin M-P, G Damasse, M Chenard, C Dionne, M Duclos, RW Bouchard, R Laforce

**Affiliations:** 1 Faculty of Medicine, Laval University, Quebec City, QC, Canada; 2 Clinique Interdisciplinaire de Mémoire, Centre Hospitalier Universitaire (CHU) de Québec, Quebec City, QC, Canada

**Keywords:** Alzheimer’s disease, Amyloid-PET, Neurodegenerative disease

## Abstract

Clinical indications of amyloid imaging in atypical dementia remain unclear. We report a 68-year-old female without past psychiatric history who was hospitalized for auditory hallucinations and persecutory delusions associated with cognitive and motor deficits. Although psychotic symptoms resolved with antipsychotic treatment, cognitive and motor impairments remained. She further showed severe visuoconstructive and executive deficits, ideomotor apraxia, elements of Gerstmann’s syndrome, bilateral agraphesthesia and discrete asymmetric motor deficits. Blood tests were unremarkable. Structural brain imaging revealed diffuse fronto-temporo-parietal atrophy, which was most severe in the parietal regions. Meanwhile, FDG-PET suggested asymmetrical fronto-temporo-parietal hypometabolism, with sparing of the posterior cingulate gyrus. A diagnosis of possible corticobasal syndrome (CBS) was made. Amyloid-PET using the novel tracer NAV4694 was ordered, and revealed significant deposition of fibrillar amyloid (SUVR 2.05). The primary diagnosis was CBS with underlying Alzheimer pathology and treatment with a cholinesterase inhibitor was initiated. Determination of underlying pathological CBS subtype is not simple even when based on extensive investigation including clinical presentation, atrophy patterns on MRI, and regional hypometabolism on FDG-PET. By contrast, amyloid imaging quickly confirmed Alzheimer pathology, and allowed rapid initiation of treatment in this complex case with early psychiatric symptoms. This case study illustrates the clinical utility of amyloid imaging in the setting of atypical cases seen in a tertiary memory clinic.

## Introduction

Amyloid imaging allows in vivo detection of fibrillary amyloid plaques in the brain, a pathological hallmark of Alzheimer’s disease (AD). Although several studies have validated this technique as a diagnostic tool, its clinical indications remain unclear. The purpose of this case report was to explore how amyloid positron emission tomography (PET) can help in the diagnosis of a complex/atypical case of corticobasal syndrome (CBS), a neurodegenerative disease well-known for its heterogeneous symptomatology and underlying pathological substrates [1–3]. In the latest criteria, probable CBS is characterized by an asymmetric presentation and at least two of: a) limb rigidity or akinesia, b) limb dystonia, c) limb myoclonus, plus two of: d) orobuccal or limb apraxia, e) cortical sensory deficit, f) alien limb phenomena [4]. In addition, different cognitive deficits may coexist as a result of the underlying pathology. Recent literature has shown that CBS can be caused by four distinct pathologies: corticobasal degeneration (CBS-CBD), progressive supranuclear palsy (CBS-PSP), Alzheimer’s disease (CBS-AD) and frontotemporal lobar degeneration (FTLD) with ubiquitin-only immunoreactive inclusions, which show immunoreactivity to the TAR DNA-binding protein (CBS-TAR). Altogether, these variables make CBS a very challenging diagnosis.

## Case Report

We report the case of a 68 year-old right handed female with no past psychiatric history who was hospitalized in Geriatrics for auditory hallucinations and persecutory delusions. Her husband reported fluctuating hallucinations and mild cognitive decline over a 3-year period before she was hospitalized. Past medical history was non-contributory and all her medical conditions were treated: rheumatoid arthritis, hypertension, osteoporosis, B12 deficiency. Later in her disease, she showed severe visuoconstructive and executive deficits, which were demonstrated through an abnormal clock drawing test, disrupted proverb interpretation and a failed Luria’s three-step test. In addition, there was ideomotor apraxia (predominant on the left), bilateral agraphesthesia, and symmetrical rigidity. More recently, the patient described cramps, which appeared to be dystonia of the distal left inferior limb. Neuropsychological assessment indicated spared memory, but she showed difficulties in language (receptive and expressive) and elements of Gertsmann’s syndrome.

**Figure 1:**
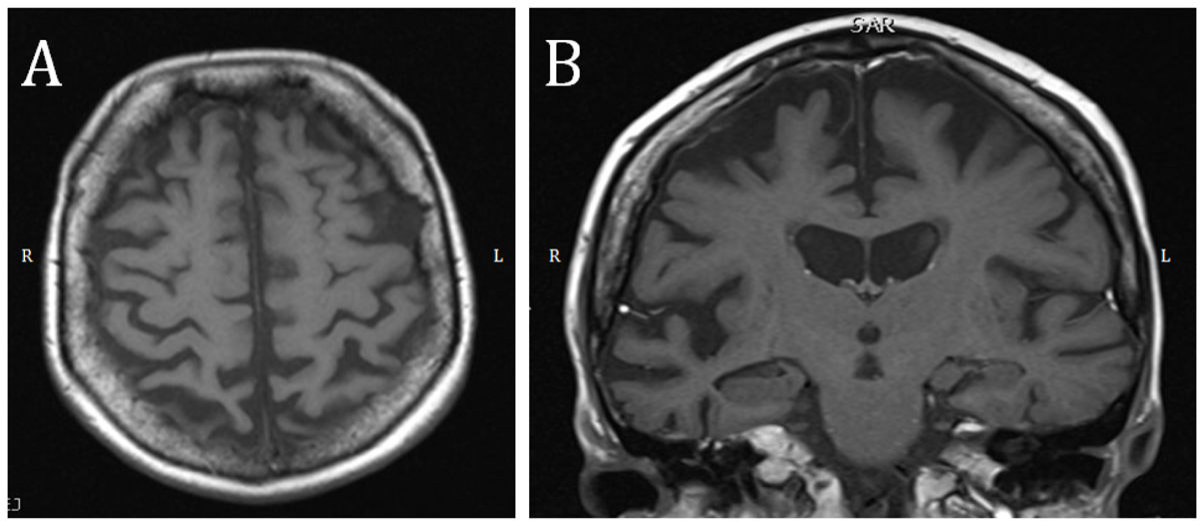
MRI of the brain. (A) Diffuse asymmetrical cortical atrophy (right>left) seen on an axial cut. The atrophy is prominent in the parietal lobes. (B) This coronal section shows that the asymmetrical cortical atrophy (right>left) extends to the posterior part of the superior temporal gyrus.

**Figure 2:**
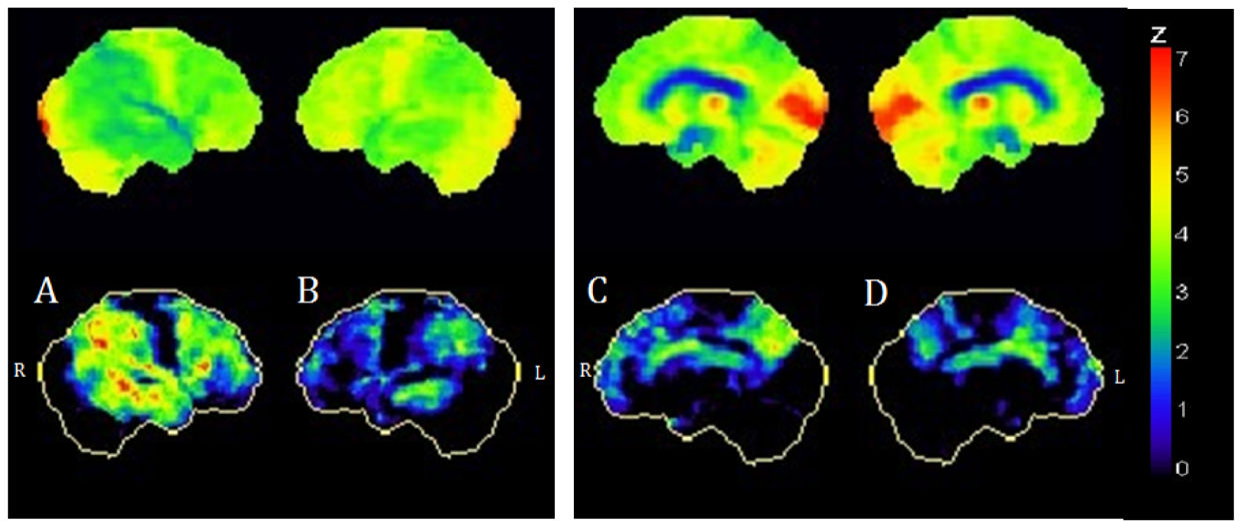
FDG-PET of the brain (A) and (B) show asymmetric bilateral fronto-temporo-parietal hypometabolism, more severe on the right while (C) and (D) show sparing of the posterior cingulate gyrus.

**Figure 3:**
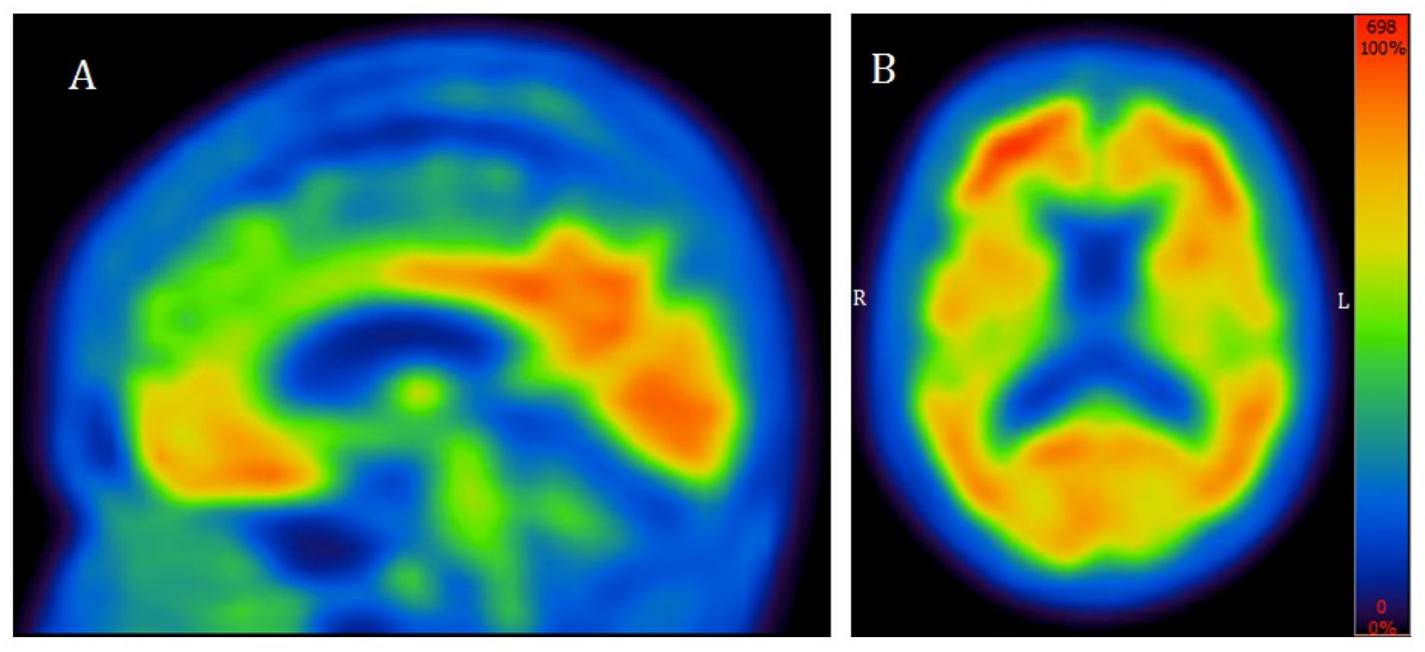
Positive amyloid-PET study using NAV4694 This test shows significant deposition of fibrillary amyloid plaques in the cingulate cortex (A) as well as diffusely throughout the brain (B).

An MRI was ordered and showed diffuse asymmetric cortical atrophy in the frontal and parietal lobes (Figure 1). An 18-Fluorodeoxyglucose (FDG) PET scan was conducted to explore the metabolic patterns (Figure 2). Finally, amyloid imaging was performed within 3 months of the FDG-PET using NAV4694 (Figure 3). This latter study was highly positive with a standardized uptake value ratio (SUVR) of 2.05 (cut-off=1.50) [5].

## Discussion

In the last few years, several studies have investigated the underlying pathologies associated with CBS [1–3]. In order of prevalence, the two most common types associated with CBS are CBD and AD [6]. Clinically speaking, a review of the literature comparing CBS-AD with CBS-CBD indicated that CBS-AD was more often associated with memory impairment (84% vs. 42%, p=0.002), cortical sensory loss (71% vs. 38%, p<0.05) and visuospatial disturbances (60% vs. 15%, p=0.009) [7]. In our case, the presence of severe visuospatial deficits and agraphesthesia suggested an AD-subtype, but memory impairment, supposedly the strongest indicator, remained surprisingly absent. The same study also attributed a lesser chance of having rigidity in CBS-AD (80% vs. 100%, p=0.02), but since a great percentage of CBS-AD present this sign, it cannot be used efficiently to discriminate between the conditions. Myoclonus is reportedly more prevalent in CBS-AD than in CBS-CBD (74% vs. 20%, p=0.0003), but was not present in our case. Finally, several authors have shown that behavioral symptoms, such as apathy, are more associated to CBS-CBD [3] but this was not found in our case. Hence, from a clinical perspective, our case remained possibly associated with CBS-AD, but with some discordant features such as the absence of memory deficits.

On the MRI of the brain, CBS-AD is associated with a predominantly posterior pattern of atrophy, involving parietal, temporal and occipital lobes [6,8]. Our case, even though no voxel-based morphometry was made, shows more severe atrophy in the parietal lobes but the atrophy is also present in the frontal lobes. A study portraying the clinical profile of PiB positive CBS showed significant atrophy in the posterior part of the left superior temporal gyrus in all cases [9]. This can be seen in our case (even though the right side is qualitatively more affected) (Figure 1). Finally, hypometabolism on FDG-PET is compatible with an AD profile, but preservation of the posterior cingulate gyrus is not (Figure 2) [10,11].

In summary, clinical and imaging findings congruent with CBS-AD are not easy to define prospectively, especially in the presence of discordant clinical and imaging elements. In the present case, amyloid imaging was positive, hence providing a tie breaker in favor of AD pathology. However, it is important to acknowledge that amyloid deposition inevitably occurs in the aging process of all cognitively normal individuals, and that a positive scan does not automatically translate into AD [12,13]. Hence, in the interpretation of positive scans, it is essential to consider every clinical and imaging element that may weigh in favor of a clinical diagnosis of AD. In this case, amyloid imaging was the deciding factor and helped initiate treatment with a cholinesterase inhibitor. Altogether, our case efficiently illustrates the value of amyloid imaging in a complex/atypical case patient seen in a tertiary memory clinic.
